# Systems Biology — the Broader Perspective

**DOI:** 10.3390/cells2020414

**Published:** 2013-06-19

**Authors:** Jonathan Bard

**Affiliations:** Department of Physiology, Anatomy & Genetics, University of Oxford, Oxford, OX1 3QX, UK; E-Mail: j.bard@ed.ac.uk; Tel.: +44-1865-315185

**Keywords:** developmental biology, evolution, graphs, mathematical, networks, phenotypic processes, physiology, systems biology concepts

## Abstract

Systems biology has two general aims: a narrow one, which is to discover how complex networks of proteins work, and a broader one, which is to integrate the molecular and network data with the generation and function of organism phenotypes. Doing all this involves complex methodologies, but underpinning the subject are more general conceptual problems about upwards and downwards causality, complexity and information storage, and their solutions provide the constraints within which these methodologies can be used. This essay considers these general aspects and the particular role of protein networks; their functional outputs are often the processes driving phenotypic change and physiological function—networks are, in a sense, the units of systems biology much as proteins are for molecular biology. It goes on to argue that the natural language for systems-biological descriptions of biological phenomena is the mathematical graph (a set of connected facts of the general form *<state 1> [process] <state 2>* (e.g., *<membrane-bound delta> [activates] <notch pathway>*). Such graphs not only integrate events at different levels but emphasize the distributed nature of control as well as displaying a great deal of data. The implications and successes of these ideas for physiology, pharmacology, development and evolution are briefly considered. The paper concludes with some challenges for the future.

## 1. Introduction

The two great lines of modern biological thinking can be see as coming from Mendel [1] and Darwin [2], who worked at about the same time, but with very different interests. Mendel, trained as a physicist, was looking for underlying simplicities when he investigated pea traits and discovered heritable “genes” that underpinned phenotypes. Darwin, always a biologist, reveled in the richness of life (encapsulated in his metaphor of *the entangled bank* with which he ends *The Origin of Species*) and realized that evolutionary change derived from the complex mix of variation, the environment and selection. Up until the molecular revolution in the middle of the last century, the Darwinian top-down approach ruled and biologists mainly saw their task as understanding the underlying complexity of life, be it as untangling physiology, discovering the tissue interactions that drove developmental change or working out how heritable change took place. The one area where the bottom-up Mendelian approach can be said to have been used in those early days was in biochemistry. 

With the discovery of the basics of molecular biology, the thrust of biological research changed and, almost overnight, the world became Mendelian. It was probably the largest paradigm shift to have occurred in science and the hunt for the simplicity of the gene dominated research for almost fifty years. It is only in the last decade or so that the mainstream biological community has lifted its collective head and properly examined the limitations of the molecular approach. It has realized that listing genes and proteins, looking for local interactions between proteins and studying what tissues expressed which genes and where they went wrong in disease was not the whole story; what was needed was a much wider picture that tried to capture the richness and complexity of the biological world. This need has been the driving force behind the rise of systems biology, the current approach to providing an integrated view of biology from genes to phenotypes—we are back in a Darwinian paradigm.

The successes of systems biology are to be seen in the textbooks of biochemistry and physiology, the scores of complex pathways that show how groups of proteins cooperate to generate a wider function (see below), and the use of differential equations to model the dynamics of these pathways and other complex phenomena, a tradition that goes back to Guyton [3], and Hodgkin and Huxley [4]) in physiology and to Turing [5] in development. These early successes are however proving difficult to follow up in other areas partly because their molecular networks are very complicated and hard to model quantitatively and partly because there have been no clear guidelines to aid us in this work.

This essay tries to pull together some of the recent work on guidelines and principles that has been emerging over the last few years and to consider how they can be used. It starts by discussing some of the principles that now appear to underpin biological systems and goes on to consider the nature of the formal language that seems appropriate for integrating the many local facts that together build up a picture that captures the richness of biological phenomena. The final part of the paper considers the implications of these views for some of the key areas of biology and where the field might be going in the next decade.

## 2. Some Basic Concepts of Systems Biology

It is probably best to start with asking the simple question *what is systems biology*? And there are of course a range of possible answers that usually include words like integration, interactions, complexity, multilevel and the like. They also include a rejection of the reductionist paradigm, which in this context roughly says that finding simple facts and working upwards in scale is the route to understanding biological complexity. This paper takes the straightforward view that systems biology is an approach to understanding how complex phenomena (e.g., developmental change, evolutionary change and physiological stability) are generated, and is based on the realization that they may involve many events at levels extending from genes and protein interactions up to the environment (the broad view of systems biology), with a particular focus on the roles of protein networks (the narrow view), and with causality being distributed. While this view is explicitly the opposite of the reductionist approach, it should be emphasized that it builds on the successes of that approach.

These successes have emphasized upwards causation: how a signal elicits a complex developmental response or how a mutation leads to disease. Systems biology does however see these successes as superficial: in development, for example, that signal only works if the responding tissue is in the correct state. Thus, for example, the early vertebrate notochord secretes sonic hedgehog which diffuses to three nearby tissues: it induces overlying neural-tube cells to become neurons, it causes adjacent somite cells to enter the pathway that leads to their becoming vertebrae; on the underlying gut however it has no effect as its cells do not express the SHH receptor (for review of this and all other aspects of development discussed here, see [6]). Similarly, while BRCA1 is often described as a gene that predisposes women to develop breast cancer, its direct roles include facilitating DNA repair and checkpoint activation [7]. A mutation in BRCA1 results in the production of mutant proteins and allows division in cells whose mitosis would normally be blocked; if the mutant proteins affect the growth pathways, the woman may get cancer. In both cases, it is simplistic to the extent of being wrong to assign causation to the protein because it is only one component in a complicated story. Systems biology reflects the search for the full story.

To say this immediately raises the question of what the full story might look like and there are two standards here, the qualitative and the quantitative. The former requires identifying all the molecular players and showing how their interactions lead to tissue behavior (typically physiological function or developmental change); the latter requires matching the outputs of quantitative models (usually formulated as a set of differential equations) to observation. It has to be said that there are very few cases outside of physiology (see below) where anything like the full, quantitative story has emerged because it is only in this area that is currently practical to measure enough rate constants and molecular concentrations.

Producing anything like even a full quantitative story is made more difficult because of the sheer number of molecular and tissue players (Table 1) and there has therefore been considerable discussion in the literature on underlying principles that can guide the search (e.g., [8,9,10]). These principles are important partly because they provide constraints on a full systems analysis of any complex biological phenomenon and partly because, at a wider level, they reflect our wish to understand how life works. The rest of this section is a discussion of some core themes that have emerged over the last few years and that were summarized in the conclusions of a recent workshop in Oxford [11,12]. 

### 2.1. Any Complex Biological Event Involves Activity at Many Levels

Levels extend from gene sequences, through molecular activity and networks to the cells, the tissues and the organism and even to the environment (Table 1), and the involvement of none can be excluded on *a priori* grounds from having a role in achieving the final result of any event from development to evolution. Complex biological phenomena turn out to involve activity at all of these levels as they inevitably include dynamic events which involve protein and metabolic kinetics together with tissue geometry, while development involves changing this geometry as well as differentiation states. 

**Table 1:** Levels and numbers in the seven-week human embryo.

Protein-coding genes	~20K
Developmental networks generating output processes (Table 2)	~60
Simple tissues	~2K *

* A simple tissue is a defined group of a single cell type with perhaps some associated matrix [13].

The unusual example of the morphogenesis of the outflow tract of the vertebrate heart demonstrates the role of these intermediate levels in directing change [14] and emphasizes that not everything in biology is immediately underpinned by genetic activity. The early outflow tract of the heart is a single, triple-layered tube with an inner epicardial layer and an outer epithelial layer; between the layers is cardiac jelly whose effect is to make the inner layer deformable and into which migrate neural crest cells. This complex tube will become two separate tubes because a septum forms in the inner tube. The driving force for septation is blood flow: such is the geometry of the heart that the two venous inflows of the heart (from the left and right horns of the sinus venosus) not only spiral distinctly as they move through the early heart but are kept separate as they move through the outflow tract. There, the haemodynamic forces generated by the two flows distort the inner tube and initiate septation in it, and this is strengthened and completed by proliferation of the neural crest cells and the eventual loss of the jelly. Morphogenesis thus involves at least four components: outflow endothelium, cardiac jelly, heart geometry and the flow of blood driven by cardiac muscle contraction. All this before one begins to think of any gene activity!

This is also one of the few examples where it is possible, in principle at least, to model morphogenesis within the framework of differential equations: the equations are those of fluid dynamics which describe the flows of the two blood streams moved by the contraction of the heart muscle, the starting and boundary conditions are essentially defined by tissue geometry. We may not know the various rate constants and tissue properties, but the system as a whole follows a clear trajectory. 

### 2.2. No Level Has Preferred Status

The advantage of the differential equation framework is that it explicitly requires the inclusion of both states (molecular concentrations and geometry) and dynamics (rates of concentration change and physical forces). In the case of outflow septation, the framework involves several tissues and their geometry as well as everything involved in the haemodynamic flow; if any one of the components is missing, development will go awry and the embryo will die. This framework does however take for granted the complex underlying molecular genetics and protein activity involved in setting up that geometry. The full range of participants includes participants at the level of genes, cells, and tissues as well as the energy flow that drives cardiac-muscle activity—and all are needed. 

This example is typical: it does not matter whether one considers an example from development, physiology, evolution or ecology, the full story always involves many participants operating at several levels [15] Even something as apparently simple as a basic biochemical network involves genes, proteins and metabolites together with some higher level properties that control the demand on the network. All are required for the system to work and if one component fails, the system fails unless there is some redundancy in the network; any such redundancy does of course add extra complexity to the system.

### 2.3. Causality Occurs Upwards, Downwards and within Levels

**Figure 1:**
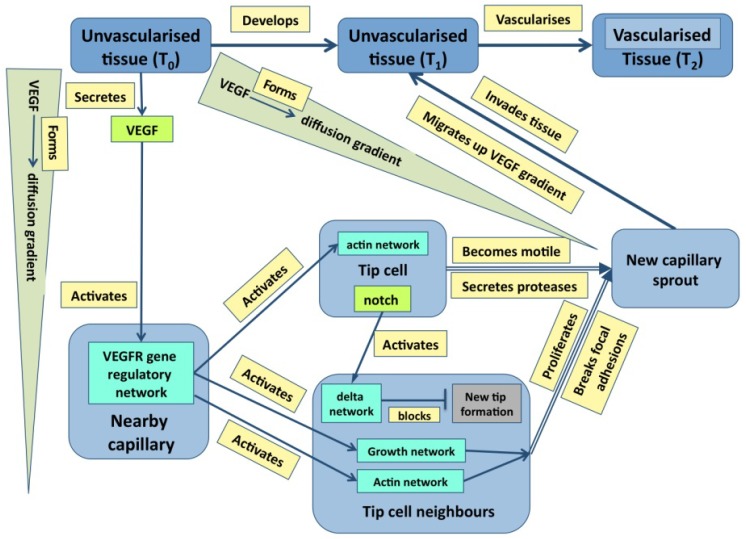
A graph describing some of the core events underpinning angiogenesis. Tissues are in blue, molecular events are in various shades of green, and processes are in pale yellow [From 35].

Just to ask where causality resides in the example of outflow septation is to realize the naivety of the question—causation is widely distributed with the haemodynamic flow, the general tissue geometry and the plasticity of the cardiac jelly all having a prime role. It is the same in cases where molecular events are more important: in the case of angiogenesis discussed later (Figure 1), it is obvious that events at many levels direct events at other level either directly or through feedback. Indeed, stability in any dynamic system normally requires negative feedback, even if it leads to oscillations about an equilibrium [16]. 

This example, by itself, negates the most simplistic view of reductionism, a straw man never really held by any biologist, that it is events at lower levels that drive change at higher, more complex levels—causation goes upwards. Since the work of Claude Bernard [17] in physiology, Darwin [2] on evolution and Monod and Jacob [18] in molecular genetics, it has been abundantly clear that there is downwards control through feedback, as well as upwards causation, and this may occur between any two levels, or even at the same level. To take an extreme case, the effects of environmental temperature on the molecular genetics of sex differentiation in crocodile embryos is well known, and there are many other cases where there is direct feedback from the environment to the genome during development [19].

One way of thinking about the location of causality in complex biological systems is to use the car engine as a metaphor: it only works if each of its components functions properly: while no component alone makes it work, there are many simple components whose failure can stop it working. So it is in biological networks: one reads in the press that scientists have discovered the gene for some disease or physiological capacity, but this is only rarely so. What has been discovered is a gene which, when mutated, blocks some capacity and so leads to a disease. Here, the resulting protein can be seen as formally equivalent to the rotator arm of a petrol engine!

### 2.4. Protein Networks Drive Biological Activity

**Figure 2:**
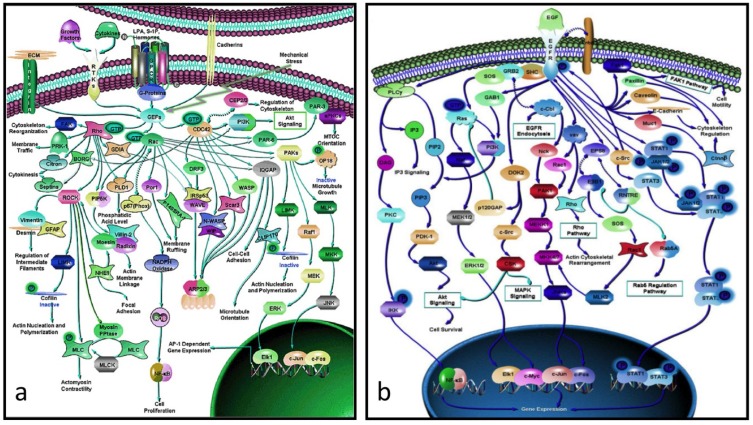
(**a**) Rho-GTPase network (> 70 proteins): an actin-activating network that underpins a wide range of cytoskeletal activity: movement, adhesion, microtubules, nuclear separation *etc.* (**b**) the EGF network (> 60 proteins) activates the cell cycle. Note that these networks are not full graphs as the details of the interactions between the proteins (the “edges”) are not specified (from [20]).

There is a considerable gap between proteins and their genes, the basic units of biological activity, and the very complex networks of proteins that drive macroscopic activity in organisms (e.g., Figure 2a,b). The former work at the molecular level interacting with one another in ways that often seem hard to comprehend. The individual protein-protein interactions are subsumed within the network as a whole, and is only when one stands back and looks at the bigger picture that one appreciates how the these interactions work together in the network to produce an output that drives some major biological property, be it for biochemistry, development, growth, physiology or even behavior.

It is clear that the role of networks in systems biology in its broader sense can be seen as equivalent to that of proteins in molecular biology, with the advantage that there are many fewer networks than proteins (most of animal development, apart from that of the neuronal system, is driven by ~60 core process networks (Table 2) some of which (e.g., those responsible for growth and microfilament activity) have properties that are tissue-dependent). This is not to underplay the detailed work of molecular biologists, but to say that they produce the trees that make up the wood. One of the triumphs of molecular biology and molecular genetics has been to produce a large number of protein networks (e.g., Figure 2 and [20]). However, a quick glance at them there shows that, in almost every case, we lack the details of the individual protein-protein relationships, and in no case do we know the rate constants of the interactions *in vivo*. Moreover, because a common result of knocking out a gene within such a network is to have no effect on the downstream phenotype, it is obvious that there is redundancy within the network. While it is usually hard to work out where this is visually, modern computational approaches are beginning to unpick these networks (e.g., [21]).

**Table 2:** Some major networks whose outputs are the processes that drive development.

Signaling	Patterning	Differentiation to	Morphogenesis	
ERK/MAPK FGF JAK/STAT Notch-delta Shh SMAD TGFβ VEGF Wnt	Hox patterning RTK patterning Notch oscillator system Signaling gradients (Shh) **Apoptosis** Caspase, fas Cellular apoptosis **Proliferation** Cyclin + down-stream events	haematopoiesis lineage erythroid lineage lymphocyte lineage myeloid lineage chondrocyte epithelium mesenchyme fibroblast muscle osteoblast neuron neuron-support cell pigment-producing cell	boundary formation	
				(eph-ephrin)
			epithelial	
				branching
				folding
				migration
				rearrangement
			mesenchymal	
				adhesion
				condensation
				migration

Note that some have outputs that reflect on/off switches (e.g., differentiation) while others (e.g., for morphogenesis) can be locally tuned [from 23].

What all this means is that, on the one hand, there is still much more work for the experimentalists to do and, on the other, that it makes some sense now for systems biologists to take a top-down view of networks and consider them as generators of function rather than becoming overly concerned about the details of how the constituent parts work. One does not need to be a mechanic to drive a car! What is worth noting is that the same networks are used in many different times and in many different places, particularly in development [6]. There are only a few signaling pathways (Table 2) that are used repeatedly, while the cell division systems and the cytoskeleton regulatory network are present in almost all cells.

### 2.5. The Genome Should be seen as Database/Resource rather than as a Formal Program

There is an obvious corollary to the idea that complex phenomena involve events distributed across many levels, with causation going downwards as well as upwards: it makes little sense to suggest that such complex phenomena derive from the execution of a single, top-down program located in the genome, or anywhere else [22]. The case for asserting that the genome holds the program for the organism’s development and function depends on assuming that, in each cell, this program sets up sensors for signals and the means for cooperating both with the like cells in its own tissue and unlike cells in neighboring tissues. Given that the only role of the genome in this context it to make new RNA sequences on demand, it is pushing the boundaries of belief too far to believe that it is helpful to see the genome as holding a program.

What makes far more sense is to view the genome as a resource, a database of coded functions that can be accessed as the system as a whole demands. Perhaps the nearest that the genome comes to storing programs is in its handling of the networks just discussed. These cell-autonomous networks (e.g., for proliferation, apoptosis, movement *etc.*) are used over and over again from development onwards and it is appropriate to view them as genomic modules or subroutines [23].

The reality of biological function is that no tissue works in isolation. Consider the development of the early metanephros which starts when a small group of cells, the metanephric mesenchyme, is invaded by the extending ureteric duct [24]. The two tissues interact through mutual signaling and, as a result, the bud undergoes a series of bifurcations which lead to its becoming the collecting-duct system while the MM undergoes the complex set of changes that leads to its producing nephrons. Here, as in most example of development, the change in the one tissue is mediated by the joint activity of signals from the other and the presence of appropriate receptors and transcription factors. The two tissues had, as part of their prior development, already synthesized their own receptors, transcription factors and signal molecules independently. The genomes in the two tissues act separately and control is distributed.

Thomas (personal communication) has suggested that perhaps the best way to view things is to consider all sub-cellular processes and the cells themselves as being part of a distributed computing system where the parts or modules communicate by way of messages. Here, there is no central “CPU”—each module can be thought of as a computational “agent” which cooperates with other agents in the system. In this approach, the biomodules would include subdomains of the genome, the networks and perhaps the geography of the cell. This type of formulation opens up the possibility of using the very rich and powerful technology of distributed and agent-based computing models. This approach, which originated with Milner [25], has led to useful languages such as the various process calculi [26,27] and holds considerable promise for systems biology. This approach can be viewed as an important alternative to differential equation-based models. Since the state space of DE models is enormous (the EGFR system, for example, has approximately 10^30^ possible states), the traditional approach cannot possibly explore it (and in any case the rate constants and concentrations are not available). It cannot, for example, easily answer questions like *Can this system produce X if A and B are inhibited*? The alternative approach (based on “model checking” techniques [28]) can answer such questions without exhaustively enumerating all the possible states.

### 2.6. the Data are Incomplete!

Perhaps the most depressing aspect of systems biology is the realization that the data is and probably always will be incomplete. If, for example, one looks at the protein network that drives cytoskeletal activity (Figure 2a) or, indeed, any other complex network, it is immediately obvious that, not only do we have no idea of the rate constants of the various interactions, but we know nothing about the details of the interactions between its protein interactions (in formal language, the edges are not annotated). Worse, many of these networks include alternate routes and it is usually not clear which subpathway might be applicable in a particular case. What this means is that we have a rough indication of what is happening but have no idea how, for example, a mutation in a particular component might affect the kinetics of the pathway or the scale of the output (e.g., the rate at which the activated EGF pathway (Figure 2b) drives the proliferation pathway). 

At the experimental level, we have few if any techniques that allow us to measure these rate constants *in vivo*, while measuring intracellular concentrations is always difficult. At the theoretical level, we often have to assume that absolute amounts of proteins are so low that the law of mass action cannot be used and stochastic descriptions have to be used [29]. As to the nature of the interactions between the proteins, much of our information comes from yeast-2-hybrid approaches and these imply an interaction between two proteins, but say nothing about the nature of that interaction. We really do need better experimental techniques here. 

## 3. The Language of Networks, Narrow and Broad

Analyzing the properties of complex protein networks such as those shown in Figure 2 is difficult. Pioneering work was done by Stuart Kaufman in the late 1960s [30] when he modeled hypothetical networks (no real ones were then known) as Boolean networks and looked for attractor states, and he and his colleagues have continued to explore these ideas in normal and cancer states [31]. There is at the moment, a considerable amount of work being done on how these networks can be analyzed [32]*.* There is also a Systems-Biology Mark-up Language [33] designed for embedding protein networks within formal programs and a Systems Biology Graphical Notation language [34] for displaying the richness of networks. 

Such protein networks describe how sets of protein interact and function, they not include their effect on the cells and tissues that contain them; they really only capture the narrow but not the broader part of the story. I have argued [35] that the basic mathematical graph or set of interconnected triplet statements of the form

<*node a*> [*edge b*] <*node c*>

provides an appropriate format for capturing the full richness of systems biology from molecules to organisms, as it is for articulating most forms of knowledge (it is noteworthy that the web is moving towards using such triplets for representing information [36]). In the context of systems biology in the broader sense, such a format has the following properties

- It can easily handle all levels from simple molecules upwards.
- It can handle upwards and downwards causality.
- It can cope with considerable amounts of complexity.
- It can distinguish between states and the drivers of state change.
- It is terse and visible on the one hand and can be easily embedded within computational models on the other.
- Any model that uses this format can easily incorporate new facts.

**Figure 3:**
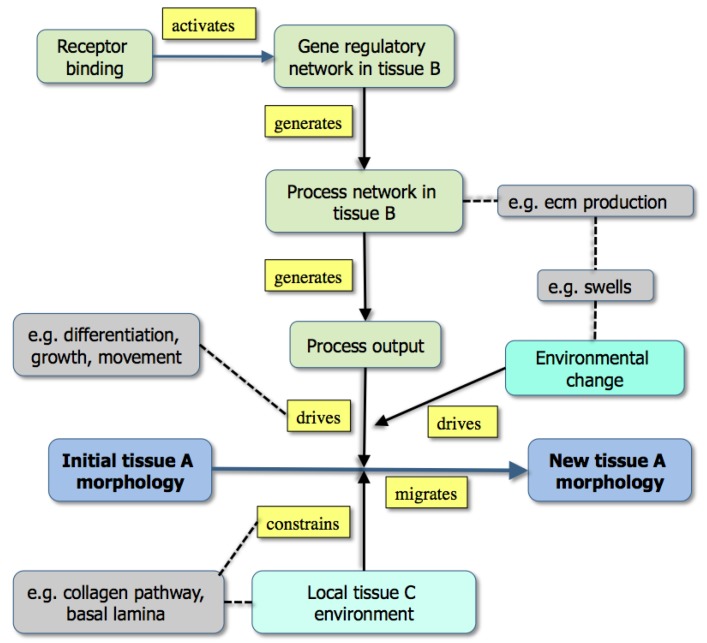
A graph showing the modeling of morphogenesis either by the downstream effects of gene activity (upper part: example [grey] is the effect of extracellular matrix production) or through existing boundary effects in the environment (lower part: example [grey box] is a collagen track used for contact guidance) [From 35].

When one considers descriptions of biological phenomena, graph statements take the form:

<*state 1*> [*process a*] <*state 2*>

where states can refer to anything from a molecule to cells to tissues and up to, for example, the environmental temperature and processes can be as simple as *interacts with* or as molecularly complex as *enters proliferation cycle*. As this approach has already been published [35], it is enough to show two diagrams here, each being the visualized expression of a set of triplet facts. The first is a general summary of how the extracellular matrix controls morphogenesis during development (Figure 3), while the second summarizes a great deal of experimental information on how capillary sprouts invade a developing tissue (Figure 1). 

While Figure 3 emphasizes that development depends not only on molecular dynamics but also on boundary conditions, the criteria mentioned above are best illustrated in Fig. 1. Here, color has been used to distinguish the different types of state (tissues, molecules, *etc*.) both to make the graph easier to follow and to emphasize the many levels of structure that it includes. It is also worth noting that single nodes can reflect whole subgraphs (e.g., the delta and the proliferation pathways). 

Such graphs have several uses in systems biology: the first is to capture in a visual and intuitively understandable way the richness of biological phenomena with their multi-level underpinnings, upwards and downwards causality and molecular complexity. Second, the triplets within an online diagram can be annotated so as to allow a user either to find the reference for the experimental data (via a Pubmed id), or to obtain further information about a gene (a uniprot id) or a tissue (an anatomical ontology id). Finally, the set of triplets provides the basis for a computational model, albeit one that may need to have added the additional and richer features of the various mark-up languages.

This is not to say that producing graphs is easy: collecting the data, making biological sense of it and organizing the triplets in a way that the graph is visually comprehensible takes time, but doing all this has the added bonus that it forces the user to think deeply about the phenomenon and he or she may identify gaps in the graph that suggest new experiments. To go back to something said earlier, producing these graphs is one way of articulating the knowledge required for both qualitative and quantitative systems models. It is of course necessary to emphasis that this knowledge produces something that is descriptive rather than executable—the latter is probably a long way down the line!

## 4. The Success of Systems Biology

The realization that we need to integrate molecular events with higher level (phenotypic) events rather than just use them as assays for molecular function (e.g., in knock-out mice) has changed biology irreversibly and, frankly, made it much more interesting. The problem is that we are still realizing that nothing in biology is as simple as it might once have seemed. The molecular complexity is daunting, partly because we have little idea of the individual roles of most proteins, partly because we do not know how they are integrated into networks and partly because so many seem to participate in generating even the simplest of functions, once one goes beyond classical biochemistry. It is fortunate that our databases, for all the information that they hold, still have plenty of space! 

In this section, I briefly review how four major areas of biology (physiology, medicine and pharmacology, development and evolution) have adopted systems approaches. Nothing is said here about neurobiology because its problems, once anatomical development has ceased, have little to do with molecular specificity and everything to do with how the same, or very similar molecular and cellular populations handle electrical signals in site-specific ways. It will however be touched on in the concluding section. 

### 4.1. Systems Physiology

The phrase is a tautology: physiology has always been about functional systems, and computational models, mainly based around differential equation descriptions, have been used for more than half a century for both neuronal and cardiac biology [3,4,37]. Recent advances in molecular genetics have however made their impact both in making models more complex and accurate on the one hand and enabling online access on the other. Here, the Physiome project [38] provides a platform for much of this work and the number of projects that can be found within its framework is impressive. In a relatively quiet way, the world physiology community has not only made available a wide variety of models for the complete range of physiological systems, but also provided modeling languages for doing this [38]. 

It is worth considering why physiology has been so successful and there are several obvious reasons. The first has been the medical imperative: understanding the dynamics of the human body is a requirement for the successful treatment of disease and there has been more than a century of well-funded support for the enterprise. Second is the relative ease with which it can be studied in vertebrates, at least: dynamic and molecular studies are the bread and butter of physiological research. Perhaps the most important reason, however, is the relative stability of the molecular and cellular systems in the functioning organism: physiologists are primarily concerned with steady state conditions, unlike developmental biologists who focus on change. While physiological systems can be very complicated (and nothing rivals the neuronal system here—see below), many of the macro-scale phenomena are well understood and some of the important protein networks are known.

### 4.2. Medicine and Pharmacology

It is becoming clear that, irrespective of the direct cause, be it a bacterium or a mutated gene, the resulting diseased state reflects abnormal behavior of molecular networks as much as the activity of a single molecule [39]. Two examples make the point. Cancers start when mutations affect the growth or apoptosis pathways or when hyperactivity in the immune system overlaps with and disturbs the normal growth pathway [40]. Congenital abnormalities such as craniosynostosis (early closure of the skull sutures) result when the balance between growth and differentiation in the early skull mesenchyme is disturbed through mutations in one or another of the key pathway components [41]. Much of contemporary work in the area of molecular medicine involves the study of disturbed biochemical and molecular-genetic networks, albeit that we have a long way to go in most cases before we understand the details of what has gone wrong.

However, where some see problems, others see opportunities! Our knowledge of how drugs work is now very detailed and the drug companies, together with academic pharmacologists, are beginning it explore where these networks overlap with those affected by disease and there are two obvious routes to success. The first comes from the realization that there are more opportunities for intervention than had previously been appreciated as we can produce novel drugs targeted against specific nodes in the networks [42]. The second uses our knowledge of drugs and the pathways that they affect: once the diseased networks have been identified, it is computationally feasible to look for overlaps between them and the networks affected by known drugs. It then becomes possible to identify a drug that may work unexpectedly or two or more drugs that can work synergistically [43]. What systems approaches to medicine are telling us is that, while the traditional drug-discovery approach of finding a magic molecular bullet to deal with the cause of a disease may have run out of steam, there are new strategies to be worked through that are as much computational as chemical.

### 4.3. Developmental Biology

While physiology is often concerned with stability, development is usually concerned with change. Although we know something about the networks that drive this change, we really only understand the key signaling pathways (Table 2). While traditional and molecular experimental approaches have allowed us to identify tissue lineages and many of the signaling interactions that drive differentiation, growth and morphogenesis, we still know little about the downstream processes that these signals initiate. Indeed, the prime wish of most developmental biologists is for a technique that would permit the identification of what lies downstream of the transcription factors that signaling pathways activate. 

This is not to deprecate the substantial advances that have been made: we know the basics of a great many networks [20] while some of the signaling pathways have been formalized as differential equations and also analyzed using graph theory [44] and process calculus [24,25]. We now have a few general models that give some insight into pattern formation (e.g., [5]), but rarely understand what is going on in any detailed way (an exception is somitogenesis (e.g., [45]). It should also be said that the main model-organism databases now hold a very large amount of molecular data about protein expression and function (much derived from transgenic technology) and this is the raw material on which systems biology builds. One point that this paper does emphasize is the core nature of developmental change: a limited set of protein networks will, once activated, generate some process (e.g., differentiation or apoptosis, Table 1), and the effect of this is to drive developmental change. Sometimes, this change can be seen as essentially cell-autonomous (e.g., differentiation); in other cases, such as the processes that drive morphogenesis, the effect of the process is constrained by existing tissues geometry. The graphs in Figure 1 and Figure 3 are intended to illustrate this. 

### 4.4. Evolution

The Modern Evolutionary Synthesis [46] suggested that forming a new species requires five steps:

1. *Mutation*. This is assumed to be through the slow accumulation of random changes to the genome.
2. *Phenotypic variation*. Genomic mutation results in anatomical and physiological changes and these are widely distributed throughout a population (e.g., the human face in all its variety).
3. *Isolation*. A small population with a skewed set of mutations that becomes isolated in some way will lead to a population that is slightly different from its parent population.
4. *Selection*. This population will thrive if it finds itself in a niche where its novel features allow it to survive, and fail if it does not.
5. *Time*. If it thrives, sufficient mutational change will eventually occur in the segregated population such that any offspring resulting from mating between the new and the original population will either fail *in utero* or will be sterile. The clearest direct evidence for this is the existence of ring species, such as the greenish warblers which form a species ring around the Himalayas [47], where neighbors can interbreed, but there is a discontinuity at a point in the ring where the variation has built up to the extent that interbreeding is no longer possible.

The key problem with this standard story of evolution is that change can be very much faster than can be accounted for by the slow accumulation of simple mutations. Darwin [2] noted how fast novel pigeon varieties could be produced by selective breeding, Waddington [48] was able to produce a strain of four-winged flies after 20 generations of ether selection that then proceeded to breed true, while the speed with which new species evolve after major extinctions is still surprising.

A key part of the explanation is that the assumption that speciation occurs through the slow accumulation of simple mutations is simply wrong; genomic change is turning out to involve major insertions, deletions and duplications as Shapiro [49] has detailed. The 1000-genome project, for example, has already shown how much variation there is in the human population [50]. What this means is that speciation within a population has far more variation within the genome to work on than the originators of the modern synthesis expected, and techniques such as high-throughput, tissue-specific proteomics techniques may well help in detailing the nature of such variation. 

Systems biology enters the story when one considers how genomic variation leads to an abnormal phenotype. This is the area of evo-devo because almost all phenotypic change in the adult organism that is subject to selection and leads to evolutionary change reflects changes that have taken place during embryogenesis. What is interesting is just how minor these changes have been in the last few hundred million years: the evolution of vertebrate tetrapods, for example, has had little effect on tissue and cell types and mainly reflects mutations in the pigmentation, epidermis, growth and secondary patterning networks (and the extent to which this generalization applies to the brain is still unclear). 

The question for systems biologists is how does mutation lead to such changes when the activity of a protein involved in a key pathway is usually indirect. We do not know the answer but can make a reasonable conjecture [51]. The dynamics of a pathway are determined partly by its constituent proteins and partly by the rate constants of its various interactions. The effect of a significant mutation on a protein-coding sequence is either to negate the function of the protein or to change the rate constants of its interactions. When one considers a complex pathway such as proliferation which can be activated by EGF (Figure 1b), the process kinetics can be affected by mutation in many proteins. The same abnormal phenotype can be produced by mutations or deletions in very different proteins. While systems biology has yet to predict a phenotype, it provides the framework within which the origin of phenotypic variation can be understood. In basic terms, the effect of mutation is to alter the output processes of networks and it is these changes, which can be amplified by mating, that lead to evolutionary change, and the idea that phenotypic change is controlled by sets of interacting proteins is one that goes back to Waddington [52]. In contemporary language, it is changes to the dynamics of process networks that drives evolutionary changes, and it is these networks that are essentially what classical evolutionary biologists view as “genes” [51].

## 5. The Challenges Facing Systems Biology

Although this paper has emphasized the broader context of systems biology, the prime challenge for the subject is straightforward and focuses on the narrow meaning of the term: it is to understand how proteins collaborate within networks to produce the processes that drive change and function. This has been done for standard biochemistry and we now need to understand the core networks of physiology and development. Once that has been done, we will not only have a far deeper appreciation of how organisms normally develop and work, but will understand how the underlying processes can go wrong and so lead to disease and to congenital abnormality. From this will come insights into how the networks can be manipulated pharmacologically to reverse abnormal function and opportunities to see how one or another drug will work on a specific genomic profile, the key step in producing personalized medicine. Further, such information is also the key to understanding how variation leads to abnormal phenotypes that are selectionally advantageous in a particular environment.

The journey will however be difficult. Just producing the diagrams of interacting proteins for these networks has been a major achievement but simple inspection shows that we have little idea as to the details of what is going one: proteins are linked by arrows but these are unlabeled and give no indication as to the nature of the interaction. We need to know these details and the rate constants before full modeling can be done. Although there are shortcuts here: fast reactions are in equilibrium long before slow ones so that effective rates of networks are decided by protracted interactions [53], it is more likely that progress in the near future will be made using computational modeling approaches [21].

The second challenge comes from neurobiology. Understanding the molecular networks that we know about should provide insight into the development of the nervous system, as this is just a branch of developmental anatomy. The specific difficulty of neuroscience is to discover the rules of how neuronal synapses are patterned and there are two separate problems: producing reflex behavior and learning. It is possible that the neuronal system of the *C. elegans* worm may prove to be the ideal model system for the former (Brenner chose this organism for just such reasons more than 50 years ago [54]), but it has to be said that it has been singularly unwilling to yield its secrets. As to the latter, we have no model organism yet, but a considerable amount of effort has been devoted to analyzing simple artificial neural networks over the last decades [55], and there is a good case for saying that it was pioneering work in systems biology, albeit that they are really metaphors for real neural circuits whose degree of complexity is still hard to appreciate.

Finally, systems biology is about ten years old and the current *zeitgeist* of biology. It is worth looking back to see what became of earlier *new areas* in biology. In the 1980s, the hot topic was molecular biology, but this has now vanished as a specialist topic as every area of biology has its own molecular basis. In the 1990s, it was bioinformatics and we all shared databases; we still do but every area of biology now has its own specialist informatics resources. It does not require much in the way of prophecy to foresee that, in the not too distant future, each area of biology will have its own multi-level approach where all levels from genes to tissues are on their way to being integrated into a single picture. Systems biologists will just be biologists and Darwin will still be king!
